# Recent Research Progress on Polyurethane Solid–Solid Phase Change Materials

**DOI:** 10.3390/polym17141933

**Published:** 2025-07-14

**Authors:** Ziqiang Wang, Jingjing Xiao, Tengkun Yao, Menghao Wang

**Affiliations:** School of Civil Engineering, Chang’an University, Xi’an 710064, China; wangzq1231@outlook.com (Z.W.); y475265458@163.com (T.Y.); 15265341659@163.com (M.W.)

**Keywords:** polyurethane solid-solid phase change materials, modification strategy, application progress

## Abstract

Research on phase change materials (PCMs) is booming in the context of global energy structure transitions and the challenge of dealing with temperature fluctuations in engineering materials. Polyurethane solid–solid phase change materials (PUSSPCMs) show great potential for thermal energy storage and temperature regulation because of their designable molecular structure, no risk of leakage, and high bulk stability. In this paper, the recent research progress on PUSSPCMs is systematically reviewed. Starting from the material system, the core preparation process of the PUSSPCMs was elucidated. At the performance improvement level, related performance studies on PUSSPCMs are systematically summarized, focusing on the introduction of dynamic covalent bonds and a nanofiller composite strategy to enhance the thermophysical properties of the materials. At the application level, innovative studies and thermomodulation advantages of PUSSPCMs in different fields are summarized. Finally, for green development, multifunctionalization, and bottlenecks in the scale-up preparation of PUSSPCMs, future research directions for balancing the performance requirements, conducting multi-scale simulations, and exploring green materials are proposed to provide theoretical references for the development and application of high-performance PUSSPCMs.

## 1. Introduction

With the accelerated transition of the global energy structure to clean and renewable energy [1], the increasing depletion of traditional fossil fuel resources, and the increasingly serious environmental problems caused by them [2], coupled with the significant challenges to the performance of engineering materials owing to temperature fluctuations under the frequent occurrence of extreme weather, the research and development of highly efficient thermal energy storage and temperature control materials has become a key technological path for balancing the efficient utilization of energy, alleviating the resource crisis, and coping with the sensitivity of the environmental temperature [3,4]. PCMs are a category of functional materials that achieve thermal energy storage and temperature regulation by absorbing or releasing latent heat during phase transitions [5,6,7]. PCMs have attracted considerable attention in the fields of building energy efficiency, electronic thermal management, and solar energy storage owing to their properties of thermal energy storage and release through phase transitions [8,9,10].

**Figure 1:**
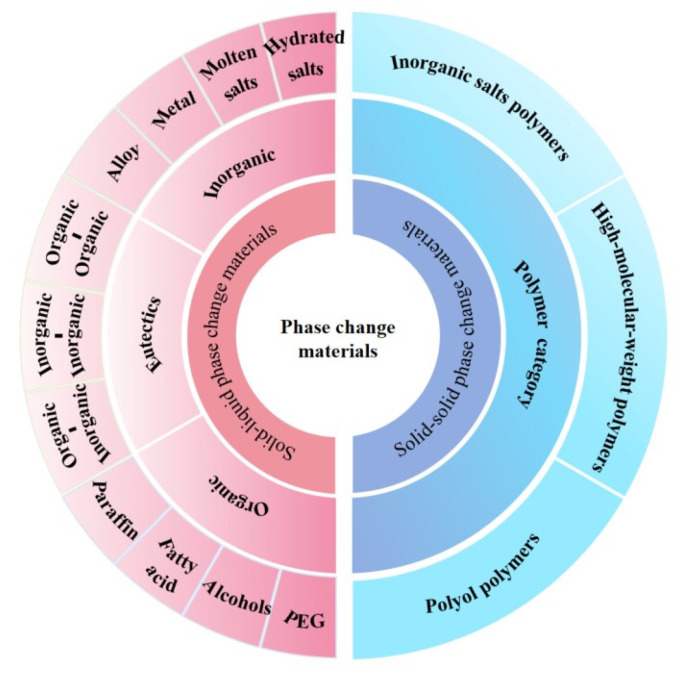
Classification of PCMs.

PCMs can be classified into four categories based on their mode of phase transition: solid–solid, solid–liquid, liquid–gas, and solid–gas [11,12]. Among them, liquid–gas and solid–gas PCMs have significant volume expansion and pressure fluctuation due to gas generation during the phase-change process, which greatly limits their engineering practicality [13]. Figure 1 shows a detailed classification of SLPCMs and SSPCMs.

**Figure 2:**
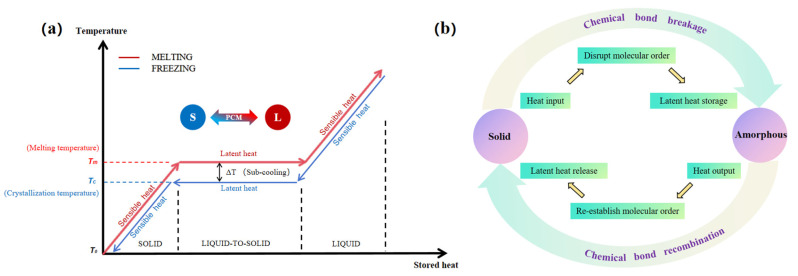
(**a**) Schematic diagram of the working of PCMs; (**b**) Phase transition process of SSPCMs.

PCMs exhibit excellent latent heat performance and high energy storage density, as shown in Figure 2a. In the heat absorption process, when the external temperature reaches the melting temperature (Tm) of the PCMs, the PCMs absorb heat through the transition of the phase state from solid to liquid, whereas in the exothermic process, when the external temperature reaches the crystallization temperature (Tc), the PCMs realize the release of heat through the transition of the phase state from liquid to solid [14,15].

For SSPCMs, the storage and release of thermal energy is achieved by the reversible crystal-amorphous form or molecular chain segment ordered–disordered transformation of phase change components (e.g., polyethylene glycol, polyol, etc.) at different temperatures [16]. Figure 2b represents this process: Their polymer backbone restricts the flow of phase-transforming substances through cross-linking or chemical bonding polymerization, ensuring that the phase-transformation process remains in the solid form [17]. During the phase transition, the molecular structure absorbs/releases the latent heat of the phase change, whereas the cross-linked network provides stability and prevents leakage of the phase change [18]. Thermally conductive enhanced fillers (e.g., GO and CNTs) optimize their heat-transfer efficiency for efficient heat storage and stable temperature control [19].

Compared with traditional sensible heat energy storage, the latent heat energy storage of PCMs has the advantages of a high energy density and small temperature fluctuation [20]. This makes it widely used in the fields of energy storage, effectively solving the problems of energy supply and demand spatial and temporal mismatch, and system temperature fluctuation [21,22,23].

**Figure 3:**
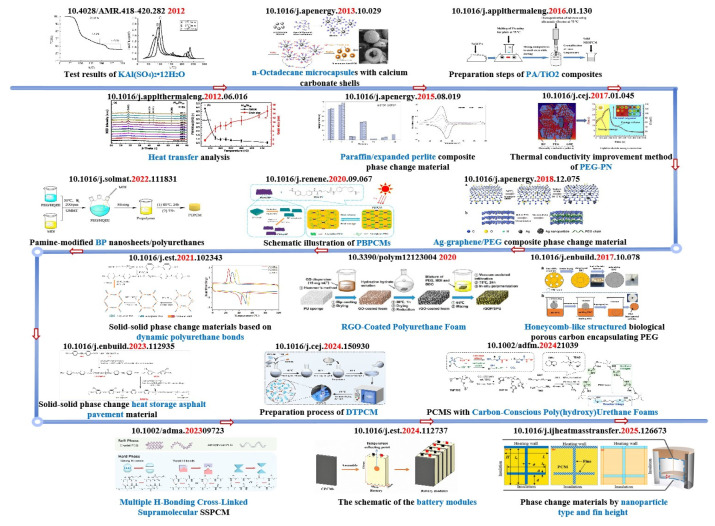
Progress of PCMs [17,29,32,33,34,35,36,37,38,39,40,41,42,43,44,45,46,47].

PCMs research was initially conducted on organic solid–liquid PCMs and inorganic solid–liquid PCMs represented by hydrated salts. However, organic PCMs and inorganic salt-type PCMs have the problems of low thermal conductivity, significant subcooling, and phase separation, respectively. In addition, the risk of liquid-phase leakage limits their long-term stability [24,25,26]. Therefore, researchers have developed composite phase change, such as microencapsulation and porous matrix adsorption [27,28]. Although these approaches alleviate the leakage problem, they introduce new challenges such as increased interfacial thermal resistance and complex preparation processes [29]. Subsequently, researchers gradually turned to the research and development of SSPCMs, the use of a crosslinking structure to enhance the stability, and the combination of nanofillers to strengthen the thermophysical properties; the current research highlights the multifunctional integration, through microcapsule encapsulation, the introduction of dynamic bonding, and composite processes to improve the performance of PCMs, to achieve the characteristics of the photo-thermal conversion, flame-retardance, self-repair, and so on [30,31]. Figure 3 shows the progress of researchers on PCMs.

PUSSPCMs have attracted considerable attention in recent years owing to the advantages of their designable molecular structure, no risk of leakage, and high volumetric stability. These advantages make these materials green. The microphase separation structure of soft and hard segments in the molecular chain provides an ideal mechanism of synergistic action between crystalline domains and flexible chain segments for thermal energy storage: the soft segments realize the storage and release of phase change enthalpy through melting and crystallization, while the hard segments maintain the overall morphology stability of the material through interactions such as hydrogen bonding. By adjusting the ratio of soft and hard segments and introducing functional monomers or chemical modification groups, the phase transition temperature, thermal conductivity, and mechanical strength of polyurethanes can be precisely adjusted to meet the needs of different applications.

In this study, the latest research progress on PUSSPCMs is systematically reviewed. This article systematically summarizes the materials required for the preparation of PUSSPCMs and the core preparation methods. It also explains how the selection of different materials affects the phase change performance of PUSSPCMs. An analysis of the advantages and disadvantages of the three preparation methods for PUSSPCMs is provided, offering a basis for subsequent selection of preparation methods. In addition, this article systematically reviews the performance improvement proposals put forward by researchers regarding the phase transition temperature, phase transition enthalpy, thermal conductivity, thermal stability, and phase change cycle performance, and conducts an in-depth analysis of the factors influencing the phase change performance. The study focused on strategies to enhance the thermal-physical properties of PUSSPCMs by introducing dynamic covalent bonds and nano-filler composite strategies, discussed the effects of introducing dynamic covalent bonds during the preparation of PUSSPCMs, and explained the performance enhancement mechanisms and potential negative impacts of nano-fillers. This paper also summarizes the innovative research and performance improvement methods for PUSSPCMs in different fields in recent years. An objective analysis of the specific challenges faced by PUSSPCMs in different application scenarios is also presented. Finally, based on the preceding analysis, four future development recommendations are proposed: performance balancing, multiscale simulations, exploration of new materials, and long-term aging performance assessments.

## 2. Preparation of PUSSPCMs

### 2.1. Overview of PUSSPCMs

**Figure 4:**
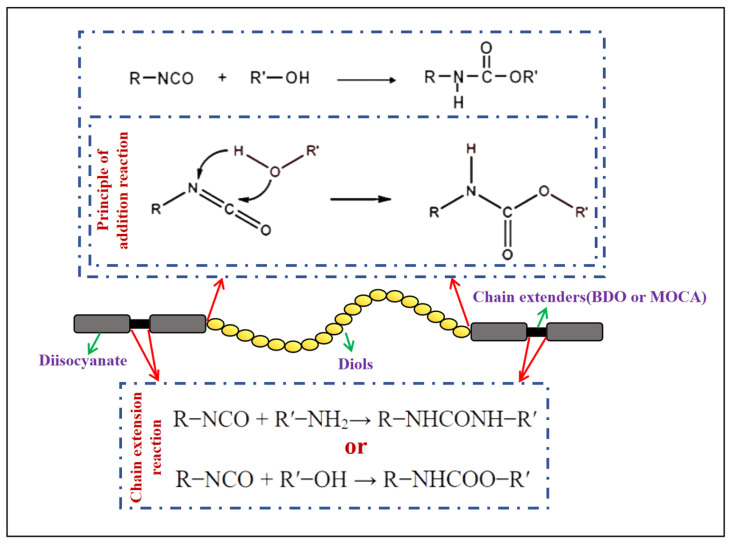
Chemical equation and introduction to polyurethane.

In recent years, PUSSPCMs have become the research focus of many scholars as important SSPCMs. PUSSPCMs are phase-change materials in which SLPCMs are chemically modified by a polyurethane synthesis process to change their phase-change behavior from solid to solid [48]. Polyurethanes are made from isocyanates (-NCO) and hydroxyl compounds (-OH) through a polymerization reaction [49]. The corresponding chemical equations are shown in Figure 4.

Polyhydroxy compounds act as soft segments in the structure of PUs, which are the working substances of PCMs. The soft segments realize the storage and release of thermal energy through reversible crystallization–melting processes in the molecular chain. The phase-change enthalpy can be regulated by adjusting the molecular weight and content of the soft segments [50]. The hard segment acts as a supporting part and maintains the macroscopic solid state of the material by forming physical cross-linking points through hydrogen-bonding interactions between the urethane groups. The crosslink density and hydrogen bond strength significantly affect the thermal stability and mechanical properties of a material [51]. Compared with SLPCMs, PUSSPCMs have a more stable phase state, which overcomes the technical bottleneck of conventional solid–liquid PCMs that are prone to leakage and require encapsulation [52,53]. PUSSPCMs remain solid throughout the phase-change process, which essentially eliminates the possibility of leakage, environmental contamination, and corrosion of equipment.

### 2.2. Materials

**Figure 5:**
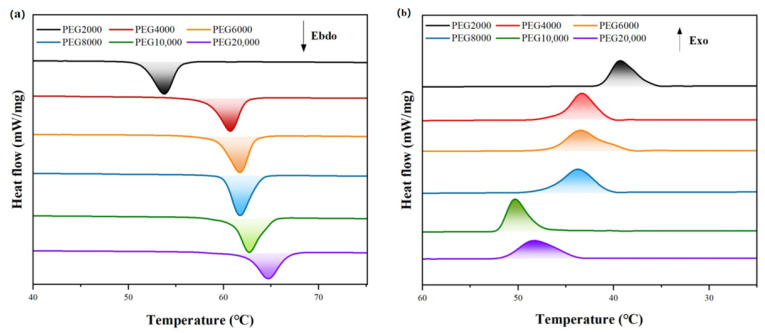
DSC curves of PEGs with different molecular weights: (**a**) endothermic process; (**b**) exothermic process.

PUSSPCMs consist of a polymer matrix, phase-change energy storage component, and functional modifier. The phase-change energy-storage part, that is, the soft segment, is mainly composed of polyols. The molecular weight and hydroxyl functionality of the soft segment can affect the phase transition temperature and enthalpy of PCMs [54]. Polyethylene glycol (PEG) and its homologs are the most commonly used soft-segment materials. This paper selects PEG with molecular weights ranging from 2000 to 20,000 to test its phase transition properties. The PEG was purchased from Shanghai, China Aladdin Bio-Chem Technology Co., Ltd. (Hong Kong) and is of analytical purity. Figure 5 shows the DSC curves of PEG with different molecular weights measured by a differential scanning calorimeter (DSC) produced by Shanghai, China TA Instruments.

Table 1 shows the melting temperature (Tm), melting enthalpy (ΔHm), crystallization temperature (Tc), and crystallization enthalpy (ΔHc) of PEG calculated from the DSC curves. The phase transition behavior of PEG exhibits regular changes regulated by molecular weight, which essentially result from the interaction between the crystallization thermodynamics and kinetics. As shown in Figure 5, the phase transition temperature increases with increasing molecular weight, with the core mechanism being the weakening of the chain-end effect. In low-molecular-weight PEG, the proportion of chain-end groups is high, disrupting the crystal lattice integrity and reducing the crystal stability. As the molecular weight increases, the proportion of chain-end groups decreases, enhancing the hydrogen bonding between molecular chains and improving the crystal lattice energy. The phase transition enthalpy exhibited a pattern of first increasing and then decreasing. In the low molecular weight range (2000–8000 g/mol), the reduction in chain-end groups and the increased mobility of chain segments synergistically promoted the ordered arrangement of molecular chains, significantly enhancing the crystallinity and increasing the phase transition enthalpy. When the molecular weight exceeded the critical value, the long-chain entanglement effect became the primary factor, hindering molecular chain diffusion, leading to a decrease in crystallinity and a subsequent decrease in phase change enthalpy. Therefore, when addressing different scenarios, it is essential to select PEG with an appropriate molecular weight as the soft segment to prepare PUSSPCMs with the desired phase-change temperature and enthalpy.

Isocyanates are hard segments of polyurethanes. The polymerization reaction of the isocyanate group with the hydroxyl group forms the backbone of the PUSSPCMs [55]. Table 2 lists several commonly used isocyanates and their respective chemical structures. The structure of isocyanates has a decisive influence on the phase-change properties of PUSSPCMs. This is primarily manifested in the following ways: aromatic isocyanates (such as MDI) have high structural rigidity, high symmetry, and strong hydrogen bonding ability, which endows them with a stronger crystallinity. The PUSSPCMs synthesized from these isocyanates exhibited higher phase change temperatures and enthalpy values, whereas more flexible aliphatic isocyanates (such as HDI) were primarily used to lower the phase transition temperature and enthalpy or broaden the phase transition temperature range. Additionally, isocyanates with regular structures and moderate compatibility (such as MDI) are more conducive to forming clear microphase-separated structures, which are critical for achieving reversible and stable solid–solid phase transitions and excellent cycling performance. The type of isocyanate also affects the thermal stability and mechanical properties of the prepolymer. Therefore, when preparing PUSSPCMs, the phase-change performance of PUSSPCMs can be effectively regulated to meet specific application requirements by reasonably selecting the isocyanate structure.

Chain expanders/crosslinkers, such as butylene glycol, trimethylolpropane (TMP), or dynamic crosslinkers (e.g., Diels–Alder bond-containing monomers), are added to regulate the crosslinking density of the molecular chains, improve the thermal stability of the materials, and inhibit phase transition leakage [56,57,58]. Several commonly used chain extenders and their chemical structural formulae are listed in Table 3. The functionality and dosage of crosslinking agents are key factors in regulating crosslink density, which increases significantly with their increase. Moderate increases in the crosslink density can significantly enhance the strength, modulus, and stability of PUSSPCMs. However, an excessively high crosslink density can hinder the crystallization/melting of the phase-change segments, leading to a decrease in the phase-change enthalpy, broadening of the phase-change peaks, or even loss of the phase-change capability. Therefore, it is essential to carefully regulate the type and dosage of chain extenders/crosslinking agents to ensure mechanical stability while maximizing the retention of the core phase-change properties.

The chemical structure of PUSSPCMs indicates that they have good chemical stability and inertness, and do not easily decompose or produce harmful substances. PUSSPCMs can also be compounded with functional additives to form PUSSPCMs to enhance their properties, such as enhancing the thermal conductivity of PUSSPCMs by introducing graphene oxide (GO), multi-walled carbon nanotubes (MWCNTs), or metal oxide nanoparticles; adding expanded graphite and black phosphorus nanosheets to enhance the photothermal conversion efficiency of PUSSPCMs; and introducing phosphorus–nitrogen compounds (e.g., tetrabromobisphenol A) or nanoscale flame-retardant fillers to enhance the flame-retardant properties of PUSSPCMs [59,60,61,62,63].

### 2.3. Preparation Methods

#### 2.3.1. Two-Step Solution Polymerization Method

The two-step solution polymerization method, also known as the prepolymer method, is the most commonly used method for synthesizing PUSSPCMs. In the first step, isocyanate and polyol polymerization reactions produce a prepolymer containing the-NCO end group. In the second step, a chain extender is added to the prepolymer to extend the molecular chain. The two-step solution polymerization method often uses toluene, acetone, and N,N-dimethylformamide (DMF) as solvents. The reaction temperature of this method can be easily controlled in the presence of a solvent. This method also significantly enhances the molecular weight and chain length of polyurethane compared to PUPCMs prepared in a one-step process. Linear PUSSPCMs, cross-linked PUSSPCMs, and hyperbranched PUSSPCMs can be prepared by two-step solution polymerization with the addition of different catalysts.

**Figure 6:**
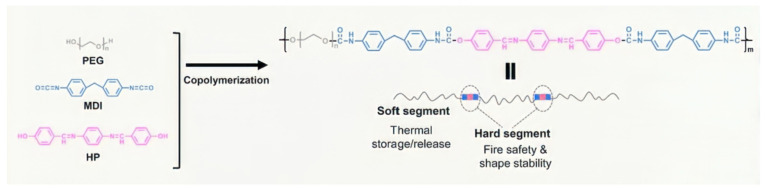
Preparation process of HPPEG-PUSSPCM [64].

Zhao PP et al. (2022) successfully prepared a novel linear polymer solid–solid phase change material (HPPEG-PU) by pre-polymerization method by dissolving PEG and MDI in DMF solution [64]. Figure 6 illustrates the preparation process.

**Figure 7:**
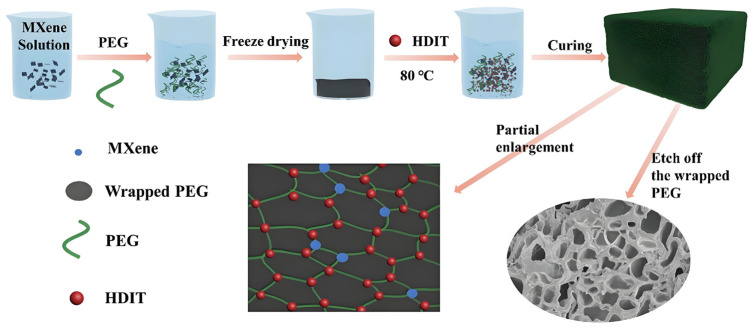
Flow chart of SSPCM preparation [65].

Gong, S. et al. (2021) constructed novel flexible polyurethane/MXene SSPCMs via a two-step polyurethane reaction (Figure 7) that significantly improved the solar thermal conversion efficiency and mechanical strength, and the formation of a three-dimensional cross-linked network consisting of HDIT (1,6-Diisocyanatohexane Trimer) and PEG conferred excellent solid–solid phase transition properties [65].

Sundararajan, S. et al. (2017) [66] introduced a hyperbranched structure by the A2 + B3 method to prepare hyperbranched polyurethane phase change materials with PEG as branched unit and mesitylene glycol (PG) as aromatic core. Its chemical structure was confirmed by FTIR spectroscopy and NMR, and it exhibited a moderately high molecular weight. Its melting enthalpy was as high as 146.6 J/g and there was no chemical degradation at 300 °C [66].

#### 2.3.2. One-Step and Ontology Polymerization Methods

The one-step polymerization method is a polymerization reaction that occurs directly between the isocyanate and polyol. This method is relatively simple and is suitable for rapid synthesis. However, the molecular structure of PU synthesized by this method is not regular, the proportion of small-molecule isocyanate and polar groups in the molecular chain of PU is small, and the restriction effect on the movement of polyol molecules is weak. Therefore, the mechanical properties and thermal stability of PU synthesized using this method are poor. The method of propriety polymerization is a polymerization reaction that does not require the presence of a medium and is carried out directly between monomers under the action of an initiator and heat. However, this method does not easily control the temperature, and as the reaction proceeds, the viscosity of the system increases, resulting in mixing difficulties.

**Figure 8:**
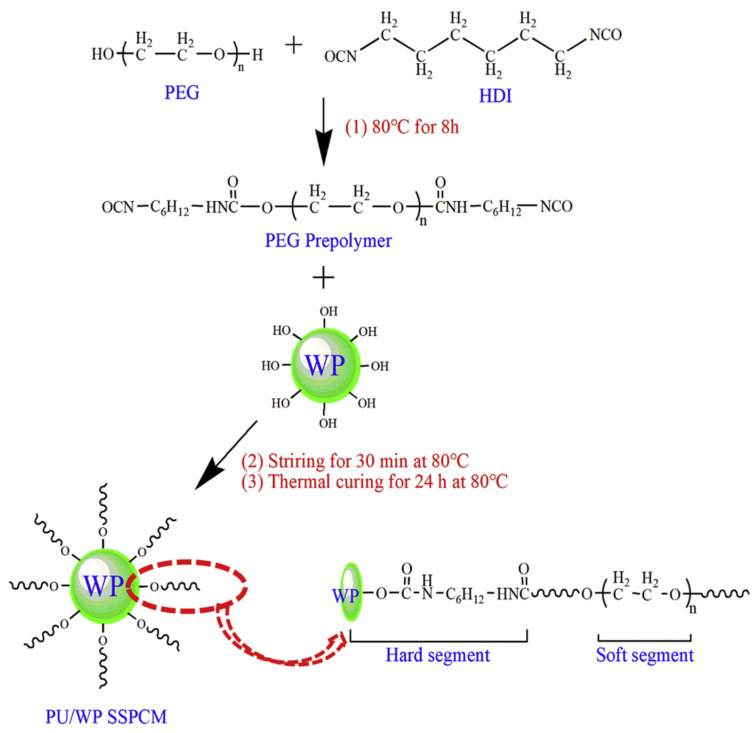
Chemical routes of PU/WP phase change materials [67].

Lu, X. et al. (2019) [67] prepared PU/wood powder (WP) SSPCMs by a one-step solvent-free method (Figure 8). The materials exhibited excellent thermal stability and photothermal conversion properties with the addition of 5 wt% HDIT content [67]. Harlé, T. et al. (2020) [54] prepared linear polyether urethanes (PUL) and cross-linked polyether urethanes (PUX) by catalyst-free synthesis. By varying the molecular weight, reaction ratio, and cross-linker content, the phase-change temperature of the PCM ranged from to 21–33 °C, the latent heat ranged from to 76–103 J/g, and the hardness of PUX was up to 30 on the shore D scale [54].

#### 2.3.3. Evaluation of the Three Preparation Methods

In the preparation of PUSSPCMs, the two-step solution method, one-step method, and bulk polymerization method exhibit significant differences in terms of molecular weight control, structural regularity, solvent use, process complexity, cost, and final material properties (such as phase-change enthalpy and thermal stability). Table 4 provides an evaluation of the three preparation methods.

As shown in Table 4, this study provides an objective evaluation of the three preparation methods. For research on phase-change performance and precise molecular structure design, the two-step solution method is the preferred choice in the laboratory; however, it must overcome solvent-related drawbacks to be effective. The one-step method has advantages in terms of the balance between performance, cost, and process simplicity, making it a commonly used method in industrial production, particularly in scenarios where phase-change enthalpy requirements are not particularly stringent and cost-effectiveness is prioritized. Bulk polymerization is an ideal choice for environmentally friendly preparation, particularly for applications sensitive to solvent residues or requiring high thermal stability. However, further improvements in the process and equipment are still needed in terms of molecular weight control, mixture uniformity, and phase-change enthalpy optimization.

## 3. Properties of PUSSPCMs

In recent years, researchers have continued to develop PUSSPCMs. Many scholars have systematically investigated the relevant properties of phase using molecular design and component optimization strategies. Table 5 presents a systematic summary of the thermophysical properties of polyurethane solid–solid phase change materials prepared by researchers for different synthetic materials in recent years.

### 3.1. Phase Change Temperature

The phase-change temperatures of PCMs include the melting temperature (Tm) and crystallization temperature (Tc). The phase transition temperature of PUSSPCMs can be targeted by using different reactants and additives and by adjusting the ratio of the hard and soft segments. The phase transition temperature of the PUSSPCMs is primarily regulated by the crystallization behavior of the soft segments. An increase in the molecular weight of the soft segments and a highly crystalline structure can elevate the phase-transition temperature and enhance the phase-transition enthalpy (ΔH). Conversely, an increase in the proportion of hard segments or strong hydrogen bonding interactions can inhibit the movement of soft segment chains and crystallization, thereby lowering the phase-transition temperature. Additionally, the introduction of strongly polar monomers (such as aromatic diisocyanates) can enhance the intermolecular forces and increase the phase transition temperature. However, excessive steric hindrance (such as side chains or rigid ring structures) can hinder the ordered stacking of segments, thereby reducing the crystallinity and phase transition temperature.

Ke, H.Z. (2017) selected five pure fatty acids, decanoic acid (CA), lauric acid (LA), myristic acid (MA), palmitic acid (PA), and stearic acid (SA), and prepared the quaternary eutectics/PU/Ag composite fibrous by using Ag nanoparticles as the support material membranes. This fiber membrane is a polyurethane-based solid–solid phase change material. The material exhibited a lower phase-transition temperature than the individual fatty acids, and the incorporation of Ag nanoparticles resulted in better thermal conductivity of the composite fibrous membranes [91]. Wei, K. et al. (2019) [92] PUSSPCMs synthesized by the prepolymerization method had a low phase transition temperature. Their low crystallinity allowed them to remain solid with no leakage during the heating process and they maintained good stability after several phase change cycles [92].

Shi, W.S. et al. (2023) synthesized PUSSPCMs by adjusting the mass ratio of PEG2000 to PEG4000. They also revealed a correlation between the phase transition temperature and the molecular structure, crystalline properties, and surface morphology of the PUSSPCMs. This correlation significantly affects the phase transition temperature of the materials [69]. Chang, J.M. et al. (2024) prepared PEG-based SSPCMs with physically cross-linked structures by blending PEGs with different molecular weights, which successfully broadened the range of phase transition temperatures to −27.4–73.2 °C [93].

The current core challenge lies in the difficulty of achieving synergy between a high ΔH, target phase-transition temperature, and stability. High-molecular-weight soft segments (such as PEG 8000) are nearing their limits; further increasing the molecular weight would lead to processing difficulties, and a high crosslinking density would significantly reduce ΔH. Low-molecular-weight soft segments exhibit weak crystallization and low ΔH values. The introduction of eutectic or disruptive monomers inevitably affects the ΔH and cycle stability of PUSSPCMs. A high ΔH requires weakened constraints to promote crystallization, whereas dimensional stability requires strengthened crosslinking, creating an inherent conflict between the two. Therefore, to achieve breakthroughs, it is necessary to develop soft segment topologies with high crystallization potential and controllable mobility or to develop new strategies for dynamically and precisely regulating microphase separation.

### 3.2. Enthalpy of a Phase Transition

The enthalpy of phase change (also called the latent heat of phase change) refers to the heat exchanged between a unit mass of material and the outside world through the process of phase transition under isothermal and isobaric conditions. The enthalpy of phase change is directly related to the heat storage capacity of PCMs. The enthalpy of phase change of PUSSPCMs is mainly determined by the crystallization capacity of the soft segment and the mass fraction of the soft segment.

However, the core disadvantage of PUSSPCMs is their significantly lower phase-change enthalpy compared to that of the corresponding SLPCMs. This is mainly because the polymer matrix itself does not participate in the phase change or contributes very little to it, occupying a portion of the material mass. The phase-change enthalpy primarily originates from the encapsulated or bonded SLPCM components. Therefore, the mass fraction of the PCM in the composite material is a key factor in determining the upper limit of ΔH.

Liu, Z.M. et al. (2017) prepared PEG-based novel stabilized FSPCM. They reduced the melt viscosity of the polycaprolactone acrylate prepolymer by adding PEG as a diluent, and the increase in PEG content conferred a high enthalpy of phase change to PCMs [83].

**Figure 9:**
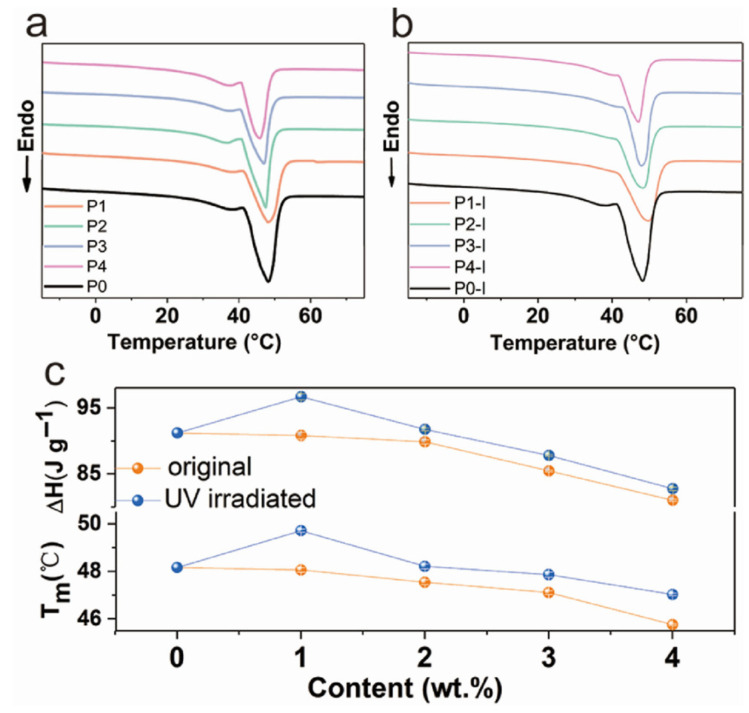
DSC curves of SSPCMs with different content of MR groups before UV irradiation (**a**) and after UV irradiation (**b**), (**c**) Tm and enthalpy of SSPCMs as a function of different MR content before and after UV irradiation [87].

Yuan, Y. et al. (2020) [87] proposed that the phase change enthalpy could be modulated by introducing photosensitive methyl red (MR) groups into PUSSPCMs. This allowed the material to exhibit increasing enthalpy and melting point (Figure 9) under UV activation [87].

Fan, X. et al. (2021) [50] synthesized PUSSPCMs with different molecular weights by adjusting the molar ratios of PEG8000, H_12_MDI, and emulsifier TS200. The enthalpy of melting and crystallization of this PCM reached 129.59 J/g and 105.45 J/g, respectively. It was also noted that the variation in molecular weight significantly affected the latent heat of the phase change and the crystalline structure [50].

Gao, N. et al. [42] (2022) prepared PUSSPCMs with excellent temperature control and thermal stability using PEG, MDI, and organo-montmorillonite (OMMT). The enthalpy of the phase change of the PCM was increased by adjusting the ratio of PEG, MDI, and OMMT [42].

Based on existing research, methods for enhancing the ΔH of PUSSPCMs primarily involve introducing components with a high ΔH_PCM and dynamic bonds. While the introduction of high-ΔH_PCM components can directly enhance efficiency, it is constrained by compatibility and the loading capacity. Dynamic bond regulation (such as Diels–Alder bonds) primarily enhances cyclic stability and may indirectly increase loading capacity, but it significantly increases costs and synthesis complexity and has a limited direct impact on enthalpy values. Breaking through the ΔH bottleneck requires progress in intelligent structural design, the development of high-performance soft segments, and precise balancing of enthalpy values with mechanical/stability properties.

### 3.3. Thermal Conductivity

**Figure 10:**
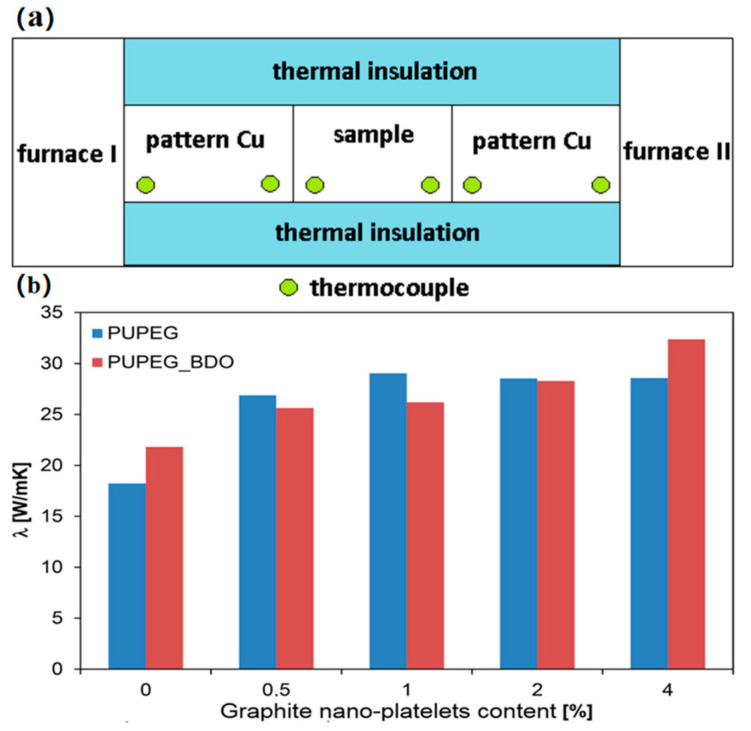
(**a**) Scheme of the thermal conductivity measurement unit; (**b**) Thermal conductivity of GNP-modified PUPEG and PUPEG_BDO [74].

PUPCMs inherently have low thermal conductivity, which limits their thermal response rate [94,95]. The low thermal conductivity of PUSSPCMs is determined by the intrinsic structure of the polymers. Polymer chains are mainly connected by weak van der Waals forces and hydrogen bonds, which are far less efficient than the regular lattices formed by free electrons in metals or the strong bonds in covalent/ionic crystals. Therefore, in the field of electronic components and solar thermal conversion, the thermal conductivity of PCMs is enhanced by introducing high-thermal-conductivity fillers, such as graphene and boron nitride [96,97]. Pielichowska, K. et al. (2016) [74] incorporated graphite nanosheets (GNP) into modified PUSSPCMs, which significantly improved the thermal conductivity of the PCMs. As shown in Figure 10, the PCM thermal conductivity increased with increasing graphite nanosheet content [74].

Mnasri, T. et al. (2018), while comparing the thermal conductivity of PCMs in composites with different configurations, found that when the PCMs were arranged in the same planes, the thermal conductivity was significantly affected by the relative position of the arrangement planes with respect to the direction of the heat flow and inlet surface [98].

Mu, B.Y. et al. (2019) synthesized rGO-PUSSPCMs, which showed a thermal conductivity of up to 0.696 W/m · K, which is far superior to those of polyether and polyurethane and a high photothermal conversion efficiency of 81.8% [99].

**Figure 11:**
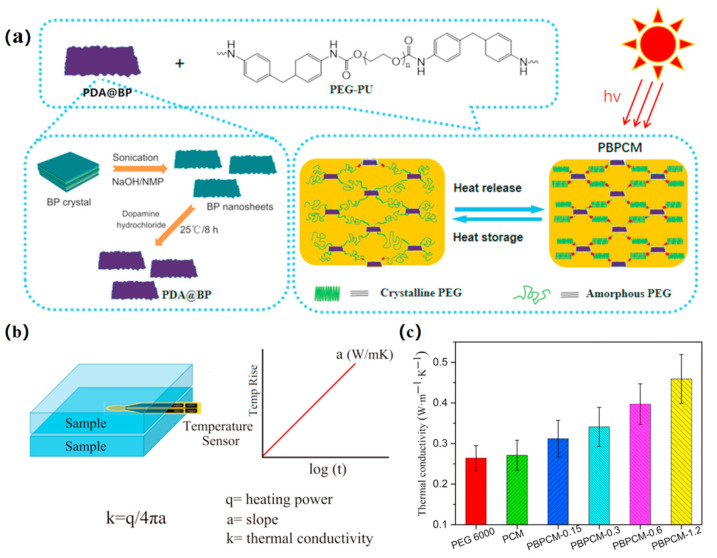
(**a**) Schematic illustration of PBPCMs; (**b**) Schematic illustration of thermal conductivity test; (**c**) Thermal conductivities of PBPCMs, PCM, and PEG 6000 [40].

Du, X.S. et al. (2021) prepared novel form-stable PCMs by compositing dopamine-decorated black phosphorus nanosheets (PDA@BP) with PEG-PU (Figure 11a). As shown in Figure 11b,c the addition of PDA@BP nanosheets increased the thermal conductivity of PEG-PUSSPCM by 69.4%, effectively reduced the amount and rate of heat release, and increased the limiting oxygen index and carbonization yield, thus significantly enhancing its flame-retardant properties [40].

Wang, J.W. et al. (2022) introduced graphene oxide (GO) into PU composites, which could significantly enhance the thermal conductivity and phase-change energy storage properties of the materials, thus effectively enhancing the solar energy storage capacity [60]. Zhang, T.T. et al. (2022) used a two-step polymerization method by using PEG as a soft segment and tubular clay nanotubes (HNTs) as a hard segment. A novel SSPCM was prepared and its thermal conductivity was enhanced by 59.0% over pure PEG by adding hexagonal boron nitride (h-BN) and graphite powder (GP) [100].

Xia, T.F. et al. (2024) formed a composite SSPCM with a synergistic cross-linked structure by constructing GO and TiO_2_ on a polyurethane framework skeleton [101]. The introduction of GO and TiO_2_ significantly improved the thermal conductivity and light absorption efficiency of the phase change material [101].

**Figure 12:**
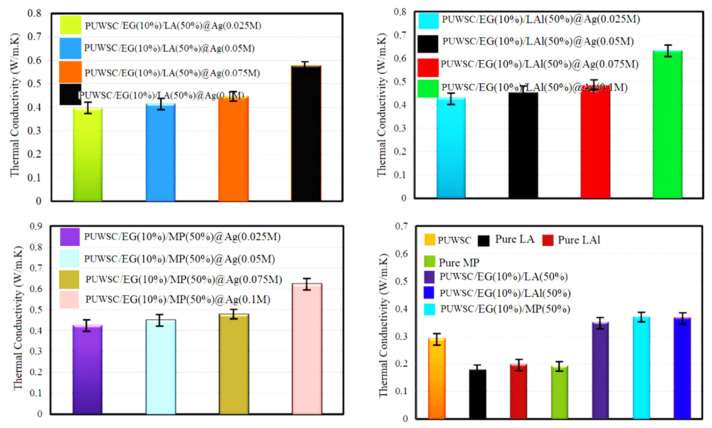
TC values for PUWSC, pure PCM, and composite SSPCM [62].

Wang, S.S. et al. (2025) suggested that the thermal conductivity of PUWSCPCMs could be significantly improved by adding expanded graphite and silver nanoparticle coatings (Figure 12), effectively preventing the leakage problem [62].

### 3.4. Thermal Stability

The thermal stability of PUSSPCMs is mainly dominated by the hard-segmented hydrogen-bonding network and dynamic cross-linking structure [102]. Aromatic diisocyanates (such as MDI and TDI) form hard segments with high-energy benzene rings and urethane bonds (-NHCOO-), whose thermal decomposition temperatures typically exceed 250 °C, serving as the primary backbone for the thermal stability of PUSSPCMs. The C-C, C-O, and C-N bonds forming the main chain of PUSSPCMs have high bond energies, requiring significant energy to break, which serves as the foundation for their resistance to thermal decomposition. Additionally, molecular groups form a three-dimensional network through moderate crosslinking, restricting the movement of molecular segments, hindering the volatilization and diffusion of thermal decomposition products, delaying the onset of thermal degradation, and slowing the degradation rate. Huang, X.L. et al. (2016) proposed that PBPEG cross-linked novel SSPCMs synthesized by hydroxyl-carboxylic condensation reaction have a phase change temperature range of 10.31–53.27 °C and a high latent enthalpy as high as 102.8 J/g, and they have a high temperature at 364 °C with good thermal stability [70]. Du, X.S. et al. (2016) synthesized comb polyurethane with polyethylene oxide segments as side chains (DMPEG-PU) with monomethoxy polyethylene glycol (MPEG), isophorone diisocyanate (IPDI) as the reactive material, BDO as the chain extender, and diethanolamine as the modifier, which exhibited satisfactory thermal stability [80].

**Figure 13:**
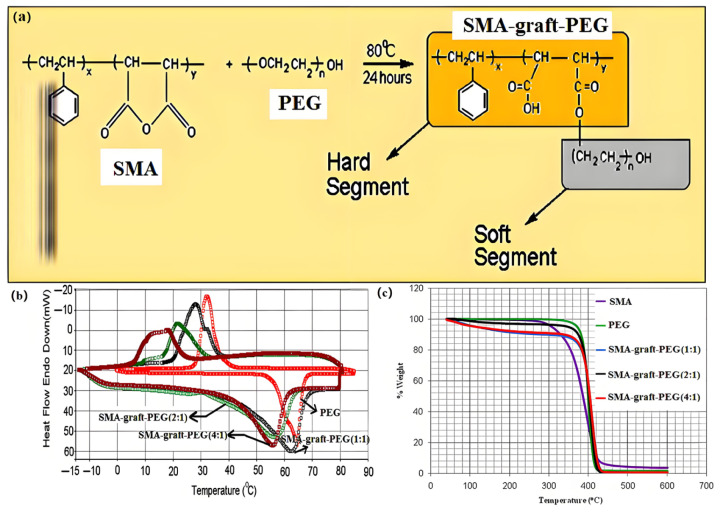
(**a**) The reaction schema regarding the synthesis of the S-SPCMs; (**b**) DSC thermograms of PEG and the synthesized copolymers; (**c**) TGA curves of the synthesized copolymers [103].

Sari, A. et al. (2017) proposed that the synthesized SMA-graft-PEG SSPCMs (Figure 13a) with latent heat storage capability has typical solid–solid phase change properties in the temperature range of 40–45 °C (Figure 13b) and possesses latent heat of phase change up to 107–155 J/g and thermal stability of at least 300 °C (Figure 13c) [103].

Zhou, Y. et al. (2018) observed that PUSSPCMs prepared by a facile solvent heat treatment showed an increase in the enthalpy of phase change and improved thermal stability in the presence of in situ-reduced graphene oxide [104].

**Figure 14:**
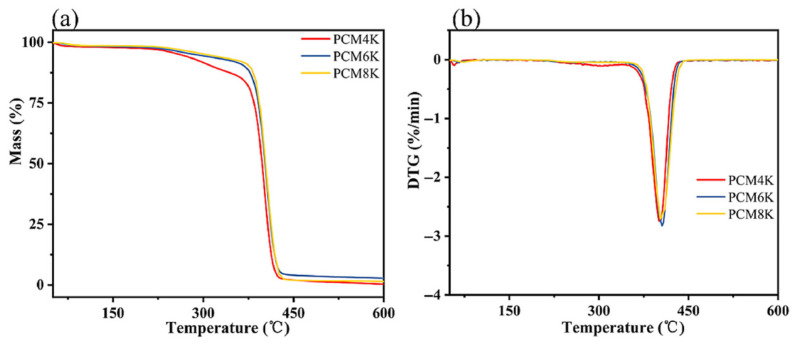
The TGA curves (**a**) and DTG curves (**b**) of prepared SSPCMs [41].

Huang, L. et al. (2021) found that SSPCM cross-linked by dynamic urethane bonding had excellent solid-state plasticity and thermal stability, which remained solid at 130 °C and had good thermal stability at 300 °C (Figure 14) [41]. Oktay, B. et al. (2021) [71] prepared a PCM by blending a PEG/octadecanol urethane phase change material with PLA. The PCM had a phase change temperature range of 10–55 °C and possessed excellent thermal stability [71].

Bai, S.J. et al. (2023) synthesized flame-retardant solid–solid phase change materials (FRPCMs) with intrinsic flame retardancy, phase change properties, self-healing, and recyclability by simultaneously introducing TBBPA and PEG into a polyurethane network backbone. This approach significantly improved the flame retardancy and thermal stability of the materials [105].

### 3.5. Phase Transition Cyclic Stability

The phase transition cycling stability is also an important property of phase transition materials. The cyclic stability of PUSSPCMs primarily stems from the confinement of the phase-change components by their crosslinked network and their lack of liquid-phase leakage. However, repeated volume changes during the phase transition process can induce microcracks, compromising structural integrity. Additionally, the slow evaporation of residual monomers, plasticizers, or phase-change monomers leads to component imbalance and a decrease in the phase-change enthalpy. These factors affect the phase-change cycle stability of PUSSPCMs.

Liao, et al. (2022) constructed SSPCM films by adjusting the molecular weight of PEG and maintained a high enthalpy of phase transition after 700 thermal cycles [106]. The HBPUSSPCMs prepared by Zhou, J.H. et al. (2023) exhibited excellent thermal stability and phase transition properties and the thermal storage capacity of the material remained stable after 500 consecutive thermal cycles [72].

Cui, M.L. et al. (2023) successfully prepared a photothermal SSPCM with tunable phase transition enthalpy and temperature by introducing a reactive photothermal agent (dihydroxynaphthalene) into a linear polyurethane SSPCM. The material exhibited long-term phase transition cycling stability and the highest efficiency of 59.7% in near-infrared photothermal conversion and 48.1% in solar photothermal conversion, and also demonstrated excellent mechanical toughness and flexibility [107].

**Figure 15:**
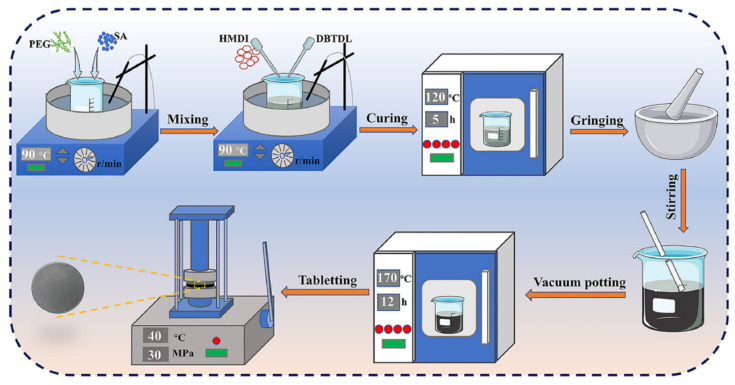
Detailed procedure diagram for preparing PU-SA/EG composites [108].

Lv, S.S. et al. (2023) constructed a biphasic polyurethane–stearic acid/expanded graphite (PU-SA/EG) composite PCM (Figure 15) with a continuous heat transport network that maintained high flexibility and thermal reliability over more than 500 temperature cycles [108].

Current research generally evaluates the phase-change cycle stability of PUSSPCMs through 100–500 cycles, which can verify short-term performance and primary failure modes but falls far short of actual application requirements. This cycle count fails to adequately reveal the impact of long-term slow processes, such as progressive component migration, cumulative low-intensity thermal degradation, filler agglomeration dynamics, and the potential degradation of self-healing efficiency. Future research urgently requires the establishment of long-term testing standards, the development of accelerated aging and lifespan prediction models, and a deeper exploration of the durability of dynamic bonds under ultra-long cycles to meet the stringent requirements of practical applications.

## 4. Design Strategies for Polyurethane-Based Solid–Solid Phase Change Composites

### 4.1. Introduction of Dynamic Covalent Bonds

In recent years, several scholars have introduced dynamic covalent bonds into PCMs to modulate the thermophysical and physical properties of phase change materials. Dynamic covalent bonding gives PUSSPCM the ability to self-heal and topologically reconfigure through a reversible fracture-reorganization mechanism. This effectively solves the problems of microcrack extension and insufficient cyclic stability of conventional materials owing to the accumulation of phase-change stresses. Table 6 summarizes the reversible conditions and chemical structures of six covalent bonds [109].

Yuan, A.Q. et al. (2020) prepared chemically cross-linked polyurea SSPCMs (OC-PCMs) with excellent thermal energy storage capacity (enthalpy up to 101 J/g) by introducing oxidized imine–amidine bonds. The OC-PCMs achieved recyclability due to the reversibility of the dynamic bonds [110].

**Figure 16:**
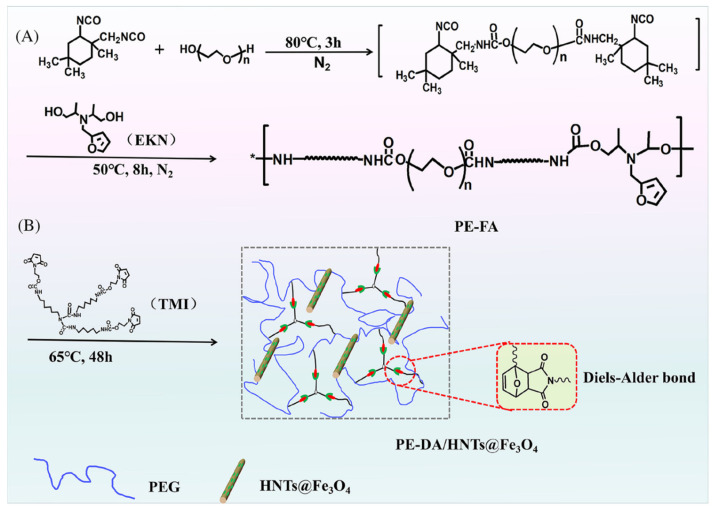
(**A**) Schematic illustration of the preparation of SSPCMs; (**B**) Introduction of Diels Alder Bonds into PCM [78].

Lin, C.H. et al. (2021), successfully prepared a novel SSPCM by introducing dynamically reversible Diels–Alder bonds (Figure 16). The material possesses excellent shape stability, thermal storage stability, and regeneration ability and effectively avoids the environmental pollution and resource waste problems of traditional SSPCMs [78].

Geng, X.Y. et al. (2024) proposed that flexible PCMs with high latent heat storage and discharge capacity, excellent toughness, and good shape memory behavior were successfully prepared by constructing a three-dimensional dynamic crosslinking network [111]. Wei, Z.K. et al. (2024) used fatty acid alcohols as the phase change constituents and introduced dynamic disulfide bonding to prepare SHPCMs. A self-healing efficiency of 99% was achieved with high energy storage efficiency [112].

Tareq, A.Z. et al. (2025) functionalized polyurethane urea oligomers by introducing hydrogen-bonded amide end-groups. This approach significantly alters the thermal, mechanical, morphological, and rheological properties of supramolecular polyurethane elastomers [113].

Huang, R. et al. (2025) [52] synthesized dynamically cross-linked Cu^2+^-OUPCMs by introducing three kinds of dynamic bonds (oxime–urethane, metal–ligand and hydrogen bonds). Such PCMs not only overcome the defects of traditional PCMs that are prone to leakage, but also possess excellent phase change properties, thermal stability, and shape stability, while simultaneously enhancing the self-healing ability of the materials and effectively extending their service life [52].

The introduction of dynamic covalent bonds has improved the performance of PUSSPCMs, significantly enhancing their cycling stability, self-healing ability, and recyclability. However, the introduction of dynamic covalent bonds may compromise the mechanical strength of PUSSPCMs, increase costs, and potentially interfere with their phase-transition behavior. Future researchers can further explore the introduction of dynamic covalent bonds by optimizing dynamic network designs to balance strength and dynamics, developing low-interference bond types and reducing the synthesis costs.

### 4.2. Introduction of Nanofillers

Nanofillers can significantly enhance the thermal conductivity of PUSSPCMs and suppress cyclic decay due to phase separation through interfacial reinforcement and three-dimensional thermal conductivity network construction. Their high specific surface area and surface modification enhance the interfacial compatibility while optimizing the mechanical strength and supercooling inhibition, which solves the problems of lagging thermal response and poor structural stability of traditional materials. The main principle is to improve the interface compatibility between the filler and polyurethane matrix and enhance the bonding strength by introducing organic functional groups through chemical grafting or physical adsorption. These advantages allow nanofillers to be widely used in phase change materials [114,115,116,117,118,119,120,121]. Table 7 summarizes the most commonly used nanofillers.

**Figure 17:**
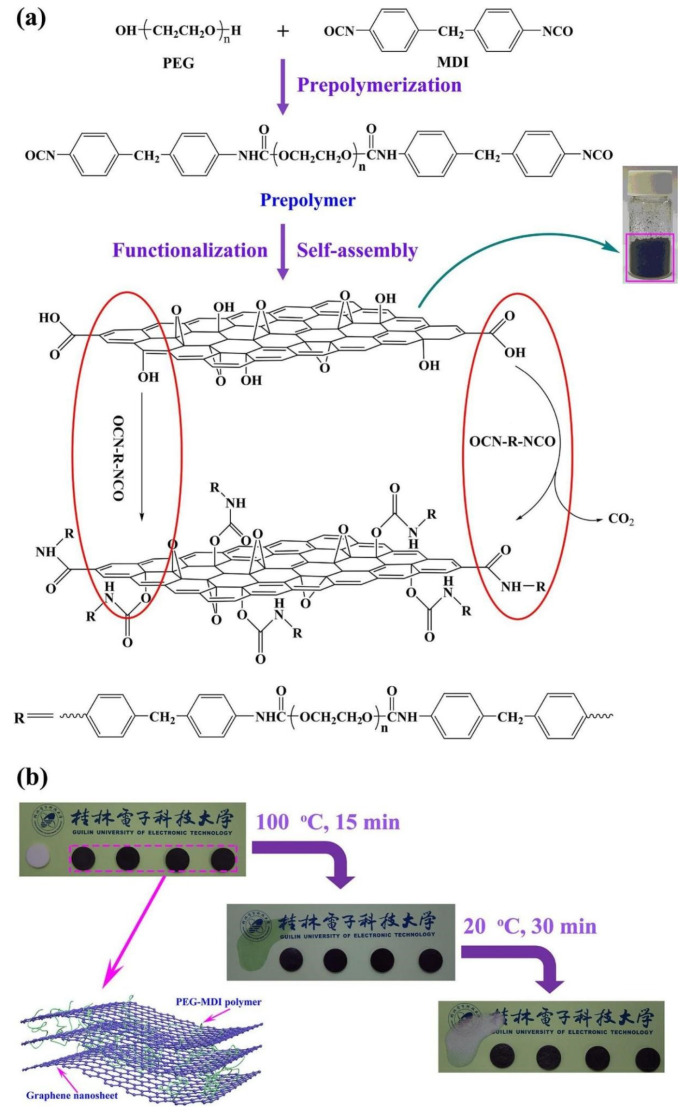
Schematic illustration for (**a**) the polyurethane prepolymer and (**b**) grafted-polymerization and self-assembly process [122].

Xia, Y.P. et al. (2019) introduced graphene oxide nanosheets, and novel polymeric SSPCMs prepared by employing self-assembly and graft-polymerization techniques (Figure 17) that exhibited excellent phase change behavior, thermal cycling stability, and high thermal conductivity [122].

**Figure 18:**
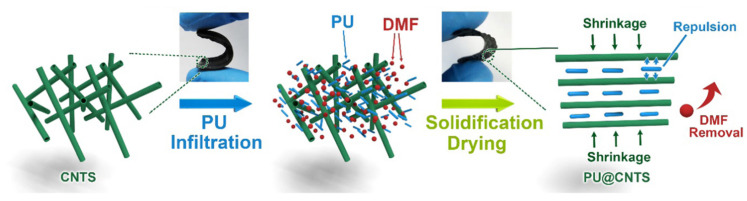
Schematic of PU@CNTS composite fabrication process [124].

Abdeali G et al. (2020) suggested that the leakage of phase change materials can be effectively reduced and their physical and thermal properties can be improved by nanostructured shape stabilization strategy [123]. Aftab, W. et al. (2019) [124] prepared the PU infiltrated CNTS (PU@CNTS) composite (PCC) (Figure 18) by infiltrating SSPCMs into carbon nanotube sponge (CNTS) pores. The nanopores enabled the PCCs to exhibit solid–solid phase change behavior. The superior carbon nanotube network resulted in an electro-optical-to-thermal energy conversion efficiency of more than 94% [124].

**Figure 19:**
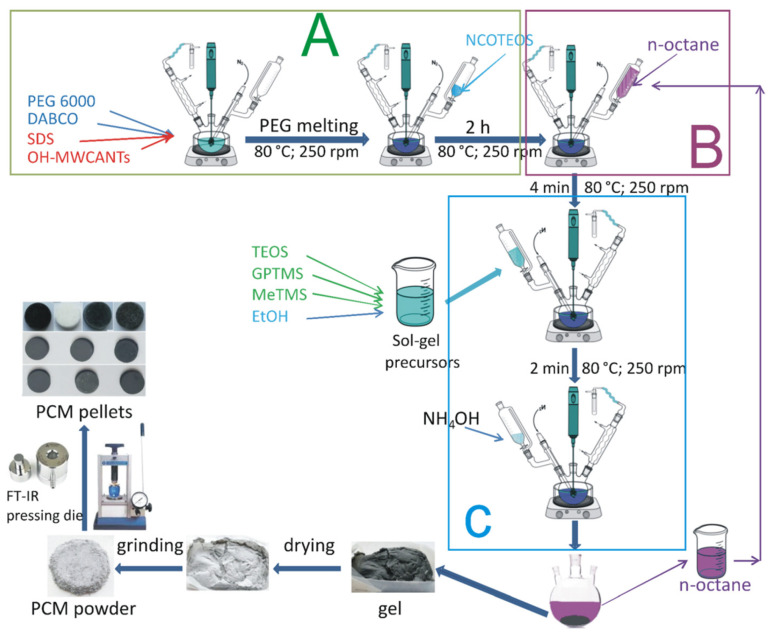
The three reaction stages (A, B, and C) of the three reaction compounds of the ssCPCMs [126].

Xiong, F. et al. (2021) successfully prepared CuS@PU Composite PCMs with excellent shape stability and thermal energy storage efficiency by doping CuS nanodiscs in a polyurethane matrix, an innovation that significantly reduced energy storage losses [125]. Nistor, C.L. et al. (2023) prepared Novel PEG6000-Silica-MWCNTs ssCPCMs with a 2/1 PEG6000/NCOTEOS molar ratio to achieve optimal stability of the PCM. Figure 19 shows the preparation of ssCPCMs. The free PEG chains interacted with the silica matrix to maintain the stability of the PCM stable. Simultaneously, the PCM maintains a high enthalpy of phase change, which significantly improves the thermal storage efficiency [126].

When introducing fillers into PUSSPCMs, the type of filler must precisely match the target performance: high-thermal-conductivity carbon-based materials or insulating ceramics are used to enhance the material’s thermal conductivity; layered materials and nano-sized phosphorus-nitrogen compounds are primarily used to enhance flame retardancy; carbon-based materials and plasma-treated metal nanoparticles confer efficient photothermal conversion capabilities on PUSSPCMs; and nano-cellulose, carbon-based materials and layered silicates are used to reinforce the material’s mechanical properties. In addition, the filler content of PUSSPCMs must be appropriately controlled. A low filler content fails to form an effective functional network, whereas a high content leads to increased agglomeration, excessive phase change enthalpy loss, and processing difficulties.

## 5. Applications of PUSSPCMs

**Figure 20:**
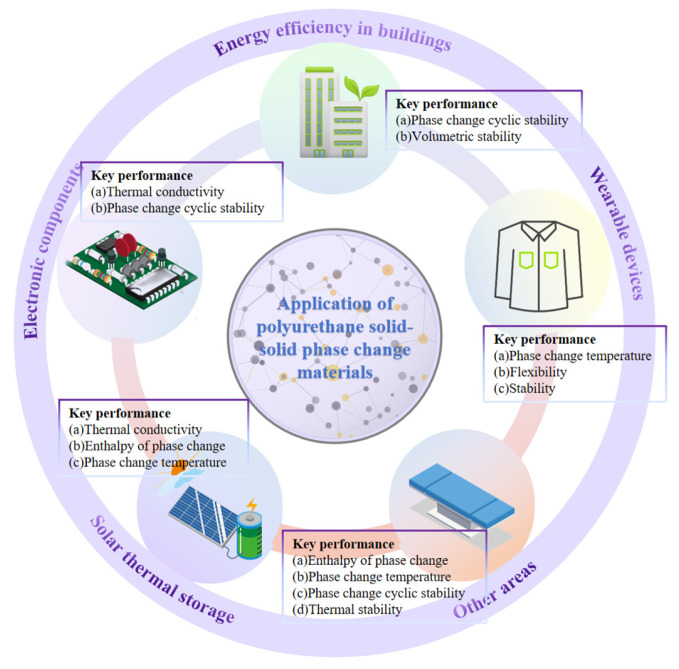
Application of polyurethane solid–solid phase change materials.

SSPCMs have received much attention in the field of thermal energy storage in recent years due to their unique advantages such as volume stability and low leakage [127]. With the development of PUSSPCMs, the structure of energy use is optimized, reducing dependence on fossil energy and carbon emissions. Combined with the studies on polyurethane solid–solid phase change materials in recent years, this chapter will summarize the research progress on the application of polyurethane solid–solid phase change materials in different fields. Figure 20 presents the main contents of this section.

### 5.1. Building Energy Efficiency

The building sector, as one of the major sources of global energy consumption and carbon emissions, accounts for more than 40% of energy consumption [128]. Among them, heat loss from the building envelope (e.g., walls, windows, roofs) is a key factor contributing to energy inefficiency. Traditional building thermoregulation relies on active heating and cooling systems, which not only consume high amounts of energy but also exacerbate the conflict between energy supply and demand. In addition, the lack of thermal inertia in building materials leads to significant indoor temperature fluctuations, which affect living comfort. Therefore, it is crucial to develop passive energy-saving technologies, particularly utilizing the latent heat storage properties of PCMs to regulate the building thermal environment.

The concept of equivalent thermal resistance established by Zingre, K.T. et al. (2020) helps in understanding the effectiveness of the thermophysical and solar radiation properties of building materials in reducing the heat gain of buildings in hot climates [129]. Arumugam, C. et al. (2024) suggested that air-conditioning energy consumption can be effectively reduced using a roofing system with integrated phase-change materials, which can satisfy building comfort needs while significantly reducing energy consumption and carbon emissions [130]. Harle, T. et al. (2022) compounded homemade SSPCMs with gypsum, which was able to increase the thermal inertia of the building, improve occupant comfort, and reduce the building’s primary energy demand [131].

**Figure 21:**
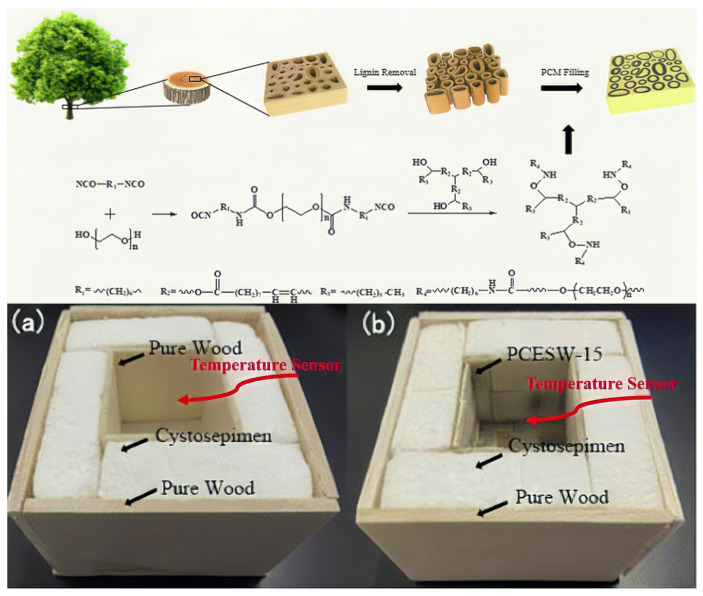
Preparation diagram and test model and the testing house models of (**a**) Pure wood and (**b**) PCESW-15 [132].

The potential value of PUSSPCMs as important SSPCMs in the construction industry has also been gradually explored. Liu, J.Y. et al. (2021) suggested that the novel phase change energy storage wood (PCESW) prepared by impregnating SSPCMs into delignified wood exhibited excellent temperature regulation performance and thermal stability. Figure 21 shows the preparation process and testing model of the PCESW. Moreover, PCESW possess excellent shape stability without liquid leakage, which provides the possibility of energy-efficient building materials for indoor temperature regulation [132].

The PLA/PU composite phase change materials prepared by Li, Z.L. et al. (2023) using the melt blending method showed excellent mechanical properties and phase change efficiency in the field of thermal energy storage. With an increase in the PLA content, the mechanical strength and sealing performance of the composites increased significantly, which is conducive to the realization of green utilization and recycling of SSPCM [133].

In addition to the application of PUSSPCMs in housing construction, research in the field of road engineering has been increasing. Under the influence of high temperatures in summer, asphalt pavements become subject to rutting and other issues, increase the heat island effect, and lead to a significant decrease in the quality and service life of asphalt pavements [134,135]. Under the influence of low temperatures in winter, the icy and snowy environments of pavements seriously affect driving safety. Against this background, researchers have gradually turned their attention to the development of phase change materials.

Wei, K. et al. (2022) [136] were able to effectively reduce the cooling rate of asphalt pavements in the region of large temperature difference and reduce low-temperature cracking by incorporating polyurethane solid–solid low-temperature phase change materials (PSLPCMs) into asphalt mixtures. The incorporation of PSLPCMs not only improves the high-temperature stability and low-temperature cracking resistance of the mixtures, but also significantly enhances their temperature-regulating ability, which results in a significant slowing of the cooling rate of asphalt mixtures [136].

Shi, J.H. et al. (2022) suggested that the incorporation of low-temperature polyurethane solid–solid phase change materials (LPSPCMs) particles into asphalt mixtures improved the low-temperature performance of asphalt mixtures and reduced the high-temperature performance and water-resistance stability [137].

Jiao, W.X. et al. (2023) suggested that by optimizing the particle size and content of PUSSPCMs, they were able to improve the temperature regulation performance of asphalt mixtures, and to reduce both the high temperature performance and the low temperature performance, with attention to their water stability [138].

**Figure 22:**
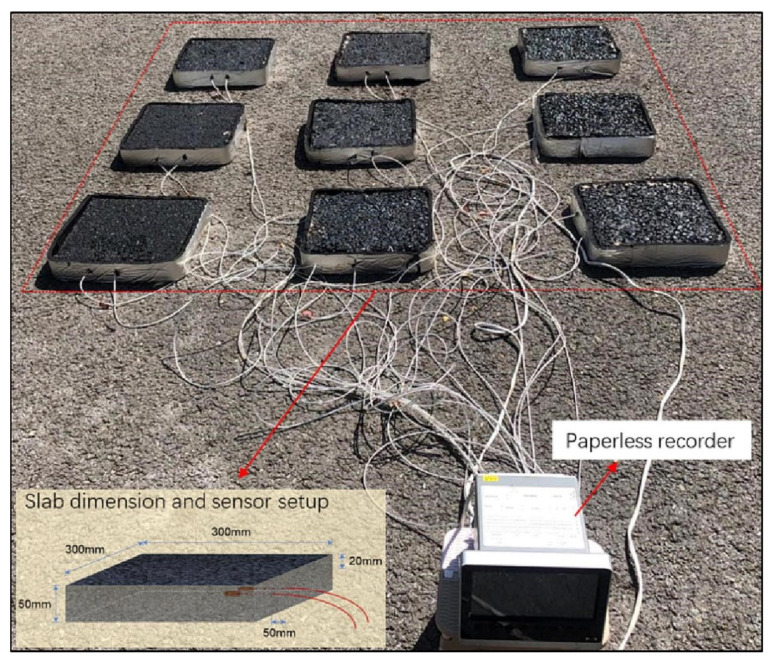
Setup for outdoor temperature regulation test of asphalt mixtures [43].

Jia, M. et al. (2022) [139] prepared novel road-use PUSSPCMs (GPCMs) using graphite as a filler through a two-step solution method. By optimizing the structure and graphite content of GPCMs, the potential of novel GPCMs as asphalt horse oil cooling fillers was significantly enhanced [139]. Jia, M. et al. (2023) [43] demonstrated a significant increase in the potential of new GPCMs as a cooling filler in asphalt horseshoe grease by replacing synthetic PUSSPCMs to replace part of the lime filler in phase change energy storage asphalt mixtures, which can effectively improve the high temperature stability and low temperature cracking resistance, while reducing the water stability. Moreover, the temperature rise rate of polyurethane-based solid–solid phase change asphalt mixtures was corroborated by outdoor experiments (Figure 22), making PUSSPCMs a potential phase-change filler [43].

Liu, T. et al. (2023) suggested that the temperature regulation properties of asphalt could be effectively improved by increasing the mass fraction of soft segments of PUSSPCMs, reducing high-temperature rutting and enhancing low-temperature cracking resistance [140]. Liu, T. et al. (2024) also suggested that the addition of PUSSPCMs and PEG4000 added to asphalt can effectively improve the thermal regulation and rheological properties of asphalt and significantly enhance its temperature regulation, resulting in more stable control of asphalt pavement temperature [141].

PUSSPCMs are widely used in the research of housing, roads, bridges, and other construction fields because of the advantages of phase-change temperature regulability, no leakage, and no corrosion. In the context of climate suitability and long-term severe service environments, the construction field has higher requirements for the phase change temperature, shape stability, durability, and phase change cycling stability of the prepared PCMs.

### 5.2. Electronics Thermal Management

Electronic devices will generate a large amount of heat owing to energy loss during operation; if the heat cannot be dissipated in time, it will lead to a sharp rise in device temperature, which in turn will lead to problems such as performance degradation, shortened life, and even thermal failure. Traditional cooling technologies have limitations such as high energy consumption and large space occupation, while solid–liquid phase-change materials can regulate the temperature through phase-change heat absorption, but there is a risk of leakage, which may lead to short-circuiting. Moreover, the small space inside electronic devices requires a higher shape stability and volume change rate of the thermal management materials.

Sari, A. et al. (2020) proposed that by optimizing the molecular structure of poly(ethylene glycol)-grafted styrene copolymer (PSAA-g-PEG), new SSPCMs with excellent latent heat of phase change and thermal stability were successfully prepared, which makes them have broad applications in the fields of building air-conditioning, thermal regulation of food packaging, automotive parts, electronic devices and solar photovoltaic panels and prospects [142].

Wu, T.T. et al. (2023) proposed that the introduction of carbon nanotube hydroxylates into PUPCMs optimizes the energy storage and mechanical properties of polyurethane phase change materials, enhances their interfacial bonding energy, and improves the thermal conductivity of the materials, which effectively reduces temperature fluctuations of the battery module during charging and discharging [143]. Luo, Y.J. et al. (2025) effectively reduced the temperature fluctuation of the battery module in charging and discharging process through the preparation of polyurethane-based flame-retardant phase change material (PMDM), which effectively reduced the heat release and leakage risk of the battery module [144].

**Figure 23:**
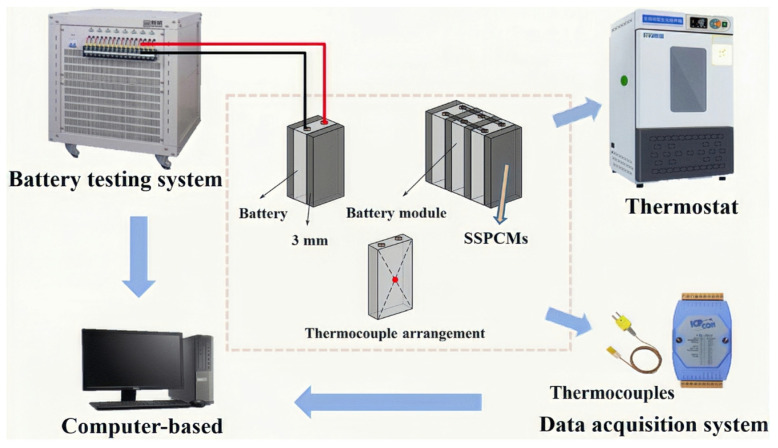
Setting up the thermal management platform [77].

Zhu, G.Y. et al. (2024) prepared novel SSPCMs by block copolymerization with excellent physical and thermal properties and no leakage problem during the phase change process [45]. Chen, M.Y. et al. (2024), chemically integrated the phase change molecular chain into a thermosetting polymer matrix, prepared novel SSPCMs that significantly improved the thermal management system (Figure 23) in terms of heat leakage resistance and mechanical integrity, and effectively reduced the leakage problem of conventional phase change materials. SSPCMs dramatically enhanced thermal conductivity, resulting in significant temperature regulation of lithium-ion battery packs at a 3C discharge rate, with internal temperature fluctuations controlled within 3 °C [77].

Salgado-Pizarro, R. et al. (2024) [145] suggested that CuC12 and MnC12 in layered hybridized organic–inorganic chalcogenides (LHOIPs) possess low thermal hysteresis and good thermal stability. This significantly reduced the thermal energy loss of SSPCMs, allowing LHOIPs to show great potential in electronic device applications [145].

**Figure 24:**
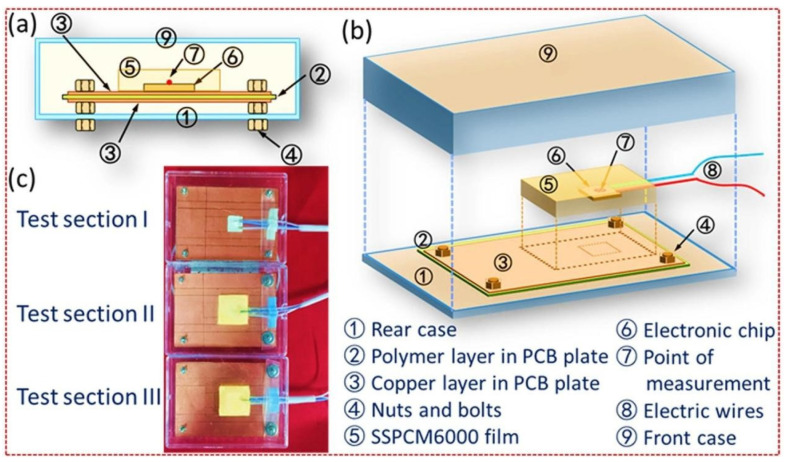
(**a**) 2D schematic and (**b**) 3D exploded view of a test section integrating SSPCM6000; (**c**) three types of section sections [146].

Liu, M.Q. et al. (2024), prepared PUSSPCMs with excellent phase transition enthalpies which were able to meet different size requirements in portable electronic devices. They provided a high-temperature alarm function for SSPCMs in smart adaptive applications through carbon nanotube thin film integration and electro-thermal conversion design (Figure 24), highlighting the important potential of SSPCMs for application in advanced technology [146].

Precision electronic components are extremely sensitive to liquid contamination. The risk of leakage from SLPCMs is an unacceptable and fatal flaw. The leak-free nature of PUSSPCMs is a key advantage for their application in electronic-thermal management. Additionally, the polyurethane matrix itself serves as an excellent insulator, eliminating the risk of circuit shorting caused by the introduction of PUSSPCMs into the circuit. The flexibility and processability of PUSSPCMs enable them to be directly molded or coated onto flexible films or gaskets, filling microscopic gaps between heat-generating chips and heat sinks. They not only provide thermal pathways but also absorb a large amount of latent heat during sudden power surges in chips, preventing rapid temperature increases and protecting the core components. This is difficult to achieve with rigid or leak-prone materials such as metals.

### 5.3. Wearable Device Thermal Management

Thermal management of wearable devices is the key to enhancing wearing comfort and functionality, but it is difficult for conventional materials to adapt to ambient temperature fluctuations, resulting in insufficient thermal comfort for the human body. Shi, J.M. et al. (2020) systematically improved the thermal storage capacity and flexibility of PU-PCMs by modulating the molecular weight of PEG, and achieved the first optimized design of PU-PCMs by weighing the flexibility and thermal storage capacity; moreover, they significantly improved the thermal conductivity and solar-driven heat conversion and storage of the composites by introducing functionalized carbon nanotubes (CNTs) performance and solar-driven thermal conversion and storage capacity, which enabled the composites to show unique potential for application in thermal management of human body parts [147].

Tian, C. et al. (2022) [148] successfully prepared SSPCMs by introducing physical cross-linking points for π-π stacking. PCMs prepared by this method were leak-free at 130 °C and possessed excellent high-temperature stability and toughness (172.44 MJ/m^3^) [148].

Zhao, Z. et al. (2022) [149] prepared a PUSSPCM fabric by nonwoven meltblown machine with excellent phase change energy storage properties. Its exothermic phase change enthalpy amounted to 60.17 J/g and the absorptive phase change enthalpy amounted to 67.09 J/g, which made the application of this fabric in clothing and tent fabrics possible [149].

Kong, M. et al. (2023) prepared smart textiles with structural stability and smart temperature control properties by assembling amorphous photonic structures and utilizing PUPCMs as binoders [150]. The TiO_2_/Cs0.33WO_3_-PU phase change material prepared by Zhang ZY et al. (2024) can effectively alleviate the heat input and subcooling problems of passively cooled materials [151].

Wu, H.Y. et al. (2024) prepared smart nanofiber cladding yarns doped with near-room temperature phase change temperature vanadium dioxide (VO_2_) by electrospinning technique, which helped to reduce the temperature of patterns on fabrics, thus reducing the large amount of energy consumed by textiles, such as garments and curtains, in the process of thermal regulation [152].

Through molecular design, the phase-change temperature of PUSSPCMs can be precisely set to the temperature required for the human skin. When the ambient temperature or skin surface temperature fluctuates, the material absorbs or releases heat through a phase change, creating a local temperature buffer layer between the skin and clothing, thereby enhancing human thermal comfort. However, the wearable device industry has high requirements for the enthalpy values of PUSSPCMs. A higher enthalpy value requires more phase-change material, which can lead to increased material thickness or weight, compromising breathability, softness, and comfort. Additionally, repeated washing, sweat exposure, UV radiation, and mechanical deformation pose significant challenges to the durability of the material.

### 5.4. Solar Energy Storage

In recent years, PUSSPCMs have been widely studied as an ideal solar thermal storage medium [153]. SLPCMs have high fluidity in the molten state, which can reduce light transmittance, corrode containers, or cause uneven distribution of photothermal materials under the influence of gravity, thereby reducing the long-term stability of photothermal conversion and heat-storage efficiency. The solid-state characteristics and good volume stability of PUSSPCMs ensure the stable dispersion of photothermal fillers in the matrix, maintaining efficient and stable photothermal conversion and heat-storage performance. Zhou, Y. et al. (2018) [154] prepared PUSSPCMs by a facile solvent heat treatment method. Owing to the synergistic effect of in situ reduced graphene oxide, the shape stability and photothermal conversion efficiency of the system were significantly enhanced. PUSSPCMs possess excellent enthalpy of phase change and thermal stability, making them promising for applications in energy storage devices such as solar energy harvesting systems [154].

Du, X.S. et al. (2020) composite Ti_3_C_2_Tx@PDA with light trapping and molecular heating into PUPCMs, resulting in a solar-thermal energy conversion and storage efficiency of 90.1% for polyurethane composite phase change materials [155].

Bao, L.H. et al. (2021) synthesized photothermal conversion polyurethane (PTPU) using cyanine dyes and PEG10000 and investigated the effects of photostability, pH sensitivity, and PEG10000 content on the properties. It was found that the cyanine dye had good photostability and was affected by pH; by adjusting the pH to 6–7, the crystallinity of PTPU increased while the thermal stability decreased. The light absorption capacity was mainly determined by the dye, the photothermal conversion efficiency and energy storage capacity of PTPU increased with increasing PEG10000 content, and the temperature under light was higher than that of the control polyurethane [156].

Li, L.X. et al. (2025) successfully prepared PEG6K-HDI-T0.6-x GO composite phase change materials with high latent heat and mechanical strength by introducing triethanolamine and intermolecular hydrogen bonding to build a strong crosslinked network. This method significantly improved the latent heat of the phase transition (101.2 J/g), flexibility, tensile strength (~35.96 MPa), and tensile strain (~1275.4%) of the materials. The SSPCMs exhibited excellent shape stability, shape memory, and self-healing properties. The addition of graphene oxide (GO) further enhanced the solar thermal energy conversion, storage, and release efficiency, resulting in a final solar thermal storage efficiency of 92% [157].

Wang, L. et al. (2025), successfully synthesized dendritic silica microspheres (DMNSiO_2_) with a connected and uniformly distributed pore structure using a novel multiblock polyurethane surfactant as a templating agent, which brings a promising application in thermal management fields such as thermal preservation materials, solar energy storage, and batteries [158].

### 5.5. Other Areas

In addition to demonstrating significant benefits in the above areas, the application boundaries of polyurethane solid–solid phase change materials are expanding. There has been a gradual increase in research in areas such as coating technology, food refrigeration, and the sensor industry.

Cobos, M. et al. (2023) showed that introducing whey protein as a green flame retardant in polyurethane dispersions with both in situ and ectopic additions significantly improved the particle size and thermal stability of the materials, which in turn enhanced their flame-retardant properties, making these whey protein–polyurethane dispersions promising for application as highly efficient flame-retardant coatings [159].

Sarkar, S. et al. (2023) [160] reviewed the application of PU-PCM composites in food refrigeration, focusing on strategies to optimize their performance in the −10~15 °C refrigeration temperature zone. By comparing the direct synthesis method with the indirect encapsulation method (EPCM), it was found that an EPCM with an organic–inorganic hybrid shell layer encapsulating a paraffin wax core could effectively solve the problems of poor thermal stability and melt infiltration, and that it combines excellent thermal reliability, matrix adhesion, thermal conductivity, and balance characteristics [160].

Kumar, A. et al. (2022) [161] prepared PEG and PU copolymer nanocomposites as flexible temperature sensors by physical mixing and chemical crosslinking. With the introduction of a carbon nanotube (MWCNT) conductive filler, the composites exhibit excellent temperature-sensitive properties [161].

## 6. Green Development of PUSSPCMs

With the growing global emphasis on sustainable development and environmental protection, research on phase change materials has gradually expanded in the direction of greening. PUSSPCMs, with their inherent advantages of no leakage and good volume stability, possess certain “green” attributes in terms of reducing the environmental risks of traditional phase change materials. However, the connotation of their green development goes far beyond this, and it is necessary to pay attention to the sustainability of raw material sources, environmental friendliness of preparation processes, and environmental benefits throughout the entire life cycle.

The raw materials for synthesizing PUSSPCMs, such as PEG and aromatic/aliphatic isocyanates (MDI, TDI, HDI, and IPDI), are all petroleum-based raw materials. To reduce dependence on fossil resources, many researchers have utilized plant-derived polyols, such as castor oil, soybean oil, and cashew nut oil, as well as lignin degradation products and sugar derivatives, as the soft segments of PUSSPCMs. Lu, X. et al. (2019) [67] prepared novel bio-based PU/WP composite PCMs. The enthalpy of melting and crystallization reached 134.2 J/g and 132.4 J/g, respectively, with a high relative enthalpy efficiency of 98.7% by introducing a WP content of 3.0 wt% [67].

The development of water-based polyurethane (WPU) greatly reduces the use of organic solvents. Hu, W.W. et al. (2021) [162] first prepared WPU@MXene aerogels with excellent elasticity by directional freeze-drying. Then, PEG was encapsulated in the WPU@MXene aerogel using the vacuum impregnation method. The WPU@MXene/PEG composite phase change material prepared by this method has an enthalpy of phase change as high as 154.6 J/g [162].

The use of solvent-free bulk polymerization in the preparation of PUSSPCMs also avoids the use of organic solvents, thereby reducing VOC emissions, energy consumption, and costs associated with subsequent solvent recovery and treatment. Kong, W.B. et al. (2017) prepared PUSSPCMs by solvent-free native polymerization with high latent heat and suitable phase change temperature, and the crystalline structure was not affected by cross-linking structure [75].

The introduction of dynamic covalent bonds endows PUSSPCMs with recyclability. Yang, Y.Y. et al. (2023) prepared cross-linked polyurethane-based photothermal phase change materials (PTPCMs) by combining p-benzoquinone dioxime (BQDO) as an endogenous photothermite with PEG. BQDO was able to confer excellent solvent resistance and mechanical toughness to PTPCMs, which enabled the PTPCMs to maintain a good anti-leakage property above the melting point of PEG. In addition, the introduced oxime-polyurethane bonds can undergo dynamic exchange reactions above the topological freezing temperature, which endows PTPCMs with recyclability [58]. This overcomes the bottleneck of PUSSPCMs recycling, which is in line with the concept of a circular economy.

In the field of building energy conservation, PUSSPCMs are used to store and release latent heat from phase changes to balance indoor temperature fluctuations, thereby reducing the energy consumption of building operations. Passive temperature regulation is a key technology for achieving carbon neutrality in the construction industry. Amaral, C. et al. (2017) suggested that by incorporating PCMs into rigid polyurethane foam (RPU), its thermal regulation could be effectively enhanced, which in turn resulted in a significant reduction in building energy consumption [163].

**Figure 25:**
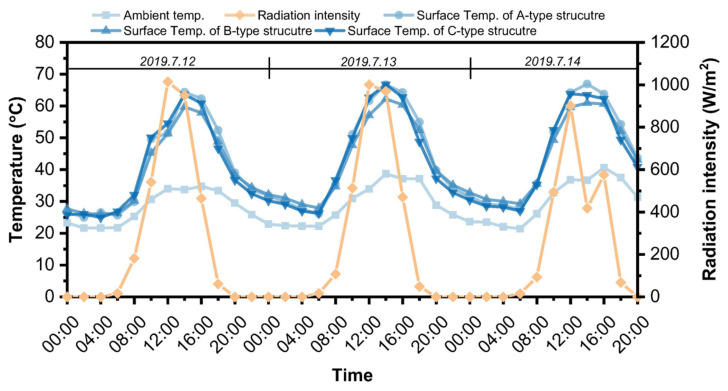
Data on surface temperatures and ambient atmospheric conditions for asphalt pavements of three different configurations [164].

Adding PUSSPCMs to asphalt pavements can effectively alleviate high temperatures on road surfaces during summer, reduce the urban heat island effect, and indirectly lower the energy consumption for cooling in cities. Sha, A.M. et al. (2022) concluded that the use of PUSSPCMs for asphalt pavement cooling can effectively mitigate the urban heat island effect and improve the functionality of asphalt pavements by comparing the data on the surface temperatures of asphalt pavements with three different structures and the surrounding atmospheric conditions (Figure 25), safety, and service life [164].

In the field of solar energy storage, PUSSPCMs can store unstable solar energy and improve solar energy utilization, thereby indirectly reducing the consumption of fossil fuels. Wang, Y. et al. (2020) [165] successfully synthesized flexible and reliable SSPCMs by integrating a PEG-based polyurethane cross-linked by double hydrogen bonds. Their high enthalpy of phase change and excellent recyclability make these SSPCMs ideal for solar energy harvesting and waste heat recovery [165].

Finally, the green development of PUSSPCMs is an inevitable trend in the future. Incorporating green concepts throughout the entire life cycle of PUSSPCMs, including molecular design, preparation, application, and recycling, is necessary for their green development.

## 7. Conclusions and Recommendations

Combined with the current research status, this paper systematically summarizes the applications of PUSSPCMs in the fields of building energy saving, thermal management of electronic devices, thermal management of wearable devices, and solar energy storage. In addition, this study focuses on summarizing the modification strategy of PUSSPCMs to solve the problems of low enthalpy of phase change and poor thermal conductivity of PUSSPCMs by introducing dynamic covalent bonding, adding nanofillers, and adjusting the ratio of soft and hard segments. Comprehensive analysis indicates that PUSSPCMs demonstrate unique advantages in thermal energy storage and management owing to their lack of leakage risk, excellent volume stability, and flexible, customizable molecular structure. However, their practical application is still constrained by significant bottlenecks, including relatively low phase-change enthalpy values, poor thermal conductivity, high raw material and preparation costs, complex multifunctional integration processes, and a lack of long-term cycling and environmental reliability data. These key challenges represent critical areas that require breakthroughs in future studies. In view of the above summary and discussion, the following possible suggestions are given for the future development of PUSSPCMs:

(a) Because PUSSPCMs are prepared by a polymerization reaction, they inherently have the problems of low latent enthalpy of phase change and low thermal conductivity. The biggest advantage of PUSSPCMs is that their phase stability results in shape stability and avoids leakage problems. Therefore, when preparing PUSSPCMs, shape stability should be considered as a prerequisite to ensure zero leakage and then combined with the needs of various fields to balance the thermal conductivity, thermal stability, and phase change cycle stability while maximizing the content of phase change substances to maximize the heat storage capacity as much as possible. Research in different fields should prioritize and optimize designs based on the core requirements of target application scenarios. For example, in the fields of electronic device heat dissipation and power battery thermal management, the top priorities should be zero leakage reliability, high thermal conductivity, and good flexibility, with the phase change enthalpy serving as a secondary optimization target in this scenario. However, in solar thermal storage systems, high phase-change enthalpy and high-efficiency solar thermal conversion capabilities are the primary design criteria. The focus should be on achieving an optimal balance among multiple performance parameters, such as phase-change enthalpy, thermal conductivity, mechanical strength, long-term thermal stability, and cost, to develop customized PUSSPCMs material systems tailored to specific application scenarios.

(b) The application of molecular dynamics simulations to reveal the microscopic phase transition mechanisms of PUSSPCMs, the nature of interactions between soft and hard segments, and the thermal transport behavior at the interface between nanofillers and the matrix provides a theoretical foundation for precise design and performance prediction at the molecular level. To optimize the performance of PUSSPCMs, multiscale simulations should be performed in the future to achieve a precise design of the molecular structure. Novel dynamic covalent bonds can be developed to better realize self-repair and performance regulation, promoting mesh to promote the research and development of multifunctional and intelligent PUPCMs. For example, the development of smart PUSSPCMs with stimulus–response characteristics, endowing materials with light-, heat-, or electrically responsive capabilities, enabling controllable phase-change triggering, on-demand heat release, or self-healing of damage, and expanding the application of PUSSPCMs in cutting-edge fields such as advanced smart thermal management and sensing.

(c) The complexity of the preparation process and the high cost of the materials required for the preparation of PUSSPCMs have limited the large-scale production of PUPCMs for practical engineering applications. In the future, it will be possible to systematically conduct life cycle assessments covering the entire process from raw material acquisition, synthesis and processing, product use, to waste disposal, thereby quantifying the environmental benefits. Simultaneously, significantly reducing material costs and achieving stable, repeatable large-scale production are considered core prerequisites for the practical application of PUSSPCMs. This can be achieved by optimizing the raw material supply chain, process routes, and scaling up production to reduce costs and improve efficiency. Addressing the issue of the environmental damage caused by the use of non-renewable raw materials, future research and development will focus on using renewable, environmentally friendly raw materials as soft segments or functional fillers. We will actively explore the synthesis pathways and application potential of challenging bio-based isocyanates to reduce the environmental footprint of materials at the source.

(d) In addition, current research lacks studies on the aging of PUSSPCMs. In the future, a unified long-term performance and reliability testing standard should be established to standardize accelerated aging test methods, such as phase-change cycle stability, thermal aging, humidity–thermal aging, and UV aging. The systematic accumulation of performance degradation data for PUSSPCMs under simulated or accelerated aging conditions should be conducted, focusing on the variation patterns of the phase change enthalpy, thermal conductivity, stability, and mechanical properties. This provides a scientific basis for predicting the service life of materials in actual engineering applications, assessing safety risks, and establishing design standards.

Although PUSSPCMs have the problems of low enthalpy of phase change, low thermal conductivity, and complicated preparation process, their excellent shape stability and structural designability advantages make them have a good prospect in the field of thermal energy storage. PUSSPCMs are expected to overcome existing performance and cost limitations and develop into a new generation of thermal management materials that combine efficient energy storage, intelligent response, environmental friendliness, and high reliability, providing important material solutions for improving energy efficiency and achieving sustainable development goals.

## Figures and Tables

**Table 1:** Phase transition temperatures and phase transition enthalpies of PEGs with different molecular weights.

Molecular Weight of PEG	Tm (°C)	ΔHm (J/g)	Tc (°C)	ΔHc (J/g)
2000	50.8	191.2	41.0	191.8
4000	57.9	201.7	45.4	200.3
6000	58.2	207.1	46.2	205.9
8000	60.0	212.1	47.3	211.2
10,000	61.3	207.4	52.1	204.2
20,000	62.5	184.7	51.9	183.6

**Table 2:** Commonly used isocyanates and their respective chemical structures.

Category	Abbreviation	Molecular Mass	Chemical Structure
Aromatic isocyanates	MDI	250.26	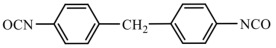
	TDI	174.16	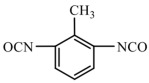
Aliphatic isocyanates	HDI	168.19	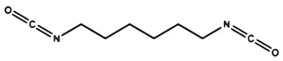
	IPDI	222.32	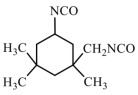
	HMDI	262.35	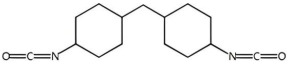
	TMXDI	244.30	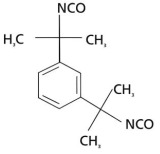

**Table 3:** Commonly used chain extenders and their chemical structure formulae.

Category	Abbreviation	Molecular Mass	Chemical Structure
Aliphatic compounds	EG	62.07	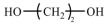
	Glycerol	92.09	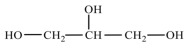
	BDO	90.12	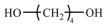
	PE/PE-T	136.15	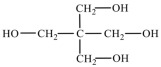
Aromatic compounds	HQEE	198.22	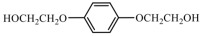
	MOCA	267.15	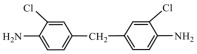

**Table 4:** Comparison table of the advantages and disadvantages of the PUSSPCM preparation methods.

Evaluation Dimensions	Two-Step Solution Polymerization Method	One-Step Method	Bulk-Polymerization
Molecular Weight Control	Excellent: Precise control over prepolymer and final product MW; Narrow MW distribution.	Poor: Typically broader MW distribution; Lower control precision.	Poor: Difficult control due to high viscosity and mixing issues; Broader MW distribution.
Structural Regularity	Excellent: Forms well-defined soft/hard segment microphase separation; Clear block structure.	Moderate: Generally lower degree of microphase separation and structural regularity compared to two-step.	Moderate: Mixing inhomogeneity may reduce regularity and affect microphase separation.
Solvent Use/Environmental Impact	Poor: Requires significant organic solvents. Issues with volatilization, recovery, and residue; Poor eco-friendliness; Residual solvent may reduce ΔH.	Variable: Can be solution-based (solvent issues similar to two-step) or bulk/solvent-free (Excellent eco-friendliness for bulk).	Excellent: Solvent-free. No issues with volatilization, recovery, or residue; Best eco-friendliness.
Process Complexity	High: Multiple steps (prepolymer synthesis, chain extension/crosslinking, solvent removal); Laborious and time-consuming.	Low: All reactants mixed and reacted in one step; Simplest process; Highly convenient.	Moderate: No solvent handling, but requires solving challenges of mixing, heat transfer, and degassing under high viscosity; Higher equipment demands.
Phase Change Enthalpy (ΔH)	High: High solvent and recovery costs; Higher energy consumption (solvent removal); Longer process time.	Low: Fewer steps, high efficiency; Lower solvent use (if used) than two-step; Lowest cost (especially bulk method).	Moderate: No solvent costs; Potentially higher equipment investment, but operational costs (energy, time) typically lower than two-step.
Cost	Typically Highest: Good structural regularity favors crystallization of soft segments; ΔH approaches theoretical value.	Moderate: Can achieve high values, but usually slightly lower than highly regular two-step products.	Variable: Depends on mixing homogeneity and structural regularity; Typically between one-step and two-step or slightly lower.
Thermal Stability	Excellent: Mild reaction conditions minimize side products; Well-defined structure generally ensures good stability.	Good: Generally sufficient for applications, depends on formulation and reaction control.	Good: Higher reaction temperatures may promote side reactions but can also form stable bonds; Absence of solvent residue benefits stability.

**Table 5:** Summary of thermophysical properties of PUSSPCMs.

Author	PUSSPCM	Melting Temperature (°C)	Crystallization Temperature (°C)	Latent Heat (J/g)	Thermal Conductivity (W/(m·K))	Thermal Stability (°C)	Phase Change Cyclic Stability	Ref.
Gao et al.	PEG + MDI + OMMT	54.97	27.35	106.8	0.3035	368 °C	-	[42]
Wei et al.	PTMEG2000 + MDI + MOCA	13.6	−3.5	49.49	0.2212	-	-	[68]
Zhang et al.	PEG6000 + MDI + BDO + SPU	42.6	41.2	61.12	0.44	-	-	[29]
Huang et al.	PEG4000 + HDI + DMG + CUCI2 + PTOL	49.8	19.5	86.67	-	325	-	[52]
Shi et al.	PEG4000 + PEG2000 + MDI + MOCA	38.45	37.53	69.38	-	230	-	[69]
Huang et al.	PEG1000 + MDI + NPG	37.32	-	120.45	-	400.1	-	[70]
Fan et al.	PEG8000 + MDI + TS200	59.08	28.47	129.59	-	300 (1.78%)	-	[50]
Oktay et al.	PEG + IPDI + n-Octadecyl alcohol	57	29	126	-	293	-	[71]
Zhou et al.	PEG8000 + IPDI + DMBA	61.34	31.66	161.57	-	>70	≥500	[72]
Zhou et al.	PEG4000 + HDIBDO	48.7–58.4	24.5–38.1	139.2	-	280–334 (5%)	-	[73]
Kinga et al.	PEG8000 + MDI + BDO	50.2–56.6	118.0–164.4	42.8	-	372–383 (5%)	-	[74]
Jia et al.	PEG8000 MDI + MOCA	50.2	111.2	32.3	-	-	-	[43]
Kong et al.	PEG8000 + PAPI	50.48	41.38	111.7	-	360	-	[75]
Jiao et al.	PEG + PTMEG + MDI + MOCA	13.6	4.1	37.1	-	-	-	[76]
Chen et al.	PEG + HDI + DBTDL	56.51	29.66	123.5	0.61	250	-	[77]
Lin et al.	PEG4000 + IPDI + EKN + TMI + FeCl_3_·6H_2_O	48.39	16.59	91.3	0.223	>250	-	[78]
Wang et al.	PTMEG + MDI + MOCA	42.8	6.1	50.7	-	>260	-	[79]
Du et al.	EPTG + IPDI + BDO	59.0	29.1	121.9	-	320–415	100 (2%)	[80]
LI et al.	PEG + MDI + CO	51.67 ± 0.20	27.04	117.9	-	>250	100 (3.3%)	[81]
Chen et al.	PEG + IPDI	46.1	40.0	80.3	3.5	280	-	[82]
Liu et al.	PEG8000 + HEA + HDDA + MDI + DBTDI	57.4	46.2	157.4	-	>200	100 (1.2%)	[83]
Wang et al.	PEG10000 + MDI + BDO + GO	57.9	40.9	152.0	0.972	379 (5%)	50 (3.8%)	[60]
Fang et al.	PEG + IPDI + DBTI + HEMA + AAO	25	-	6.49	-	-	-	[84]
Wu et al.	PEG6000 + HDI + PGF	43.8	38.9	60.3	10.86	300	-	[85]
Haung et al.	PEG8000 + HDI + HPMC	56.68	42.20	158.59	-	-	>100	[27]
Zhou et al.	PEG4000 + HDIB + I-AA + HNT + GO	57.4	33.7	103.3	-	316 (5%)	-	[86]
Yuan et al.	PEG8000 + MDI + EA + MR + DBTDI + BAH	47.9	-	87.42	-	-	-	[87]
Yang et al.	PEG6000 + IPDI + DBTDI + BDO	40.2	24.9	71.1	0.370	250	-	[88]
Lu et al.	PEG6000 + HDIT + DBTDI	63.9	33.9	89.7	-	422.9	>200	[89]
Chen et al.	PEG + MDI + BDO + n-Eicosane	57.43	-	141.2	-	-	-	[90]
Cao et al.	PEG + MDEA + MDI + BDO	55.7	33.6	137.5	-	-	-	[51]

**Table 6:** Reversible conditions and chemical structures of different covalent bonds.

Types of Dynamic Covalent Bonds	Reversible Conditions	Chemical Structure
Diels–Alder	Thermally reversible	[4 + 2] cycloaddition products
Disulfide bond	Thermal, redox, or mechanical force	–S–S–
Imine bond (Schiff base)	pH or humidity	–C=N–
Transesterification bond	Thermal or catalytic	–COO–
Hydrazone bond	pH-responsive	–NH–N=C–
Borate ester bond	pH or saccharide-triggered	–B–O–

**Table 7:** Summary of nanofillers.

Nanofiller Type	Specific Material	Function	Modification Strategies
Carbon-based Fillers	GO	Enhance thermal conductivity Improve mechanical strength	Amination (APTES) Carboxylation
	CNTs	Directional thermal conduction Suppress supercooling	Acid treatment (HNO_3_/H_2_SO_4_) PEG grafting
Ceramic Fillers	BN	High thermal conductivity Electrical insulation	Hydroxylation (H_2_O_2_) Silane coupling (KH550)
	SiO_2_	Inhibit phase separation Reduce supercooling	Hollow structure Stearic acid grafting
Metal Oxides	Al_2_O_3_	Improve thermal stability Reduce thermal expansion	Nanowire morphology Phosphate treatment
	ZnO	Photothermal conversion	Nanorod arrays Ag nanoparticle loading
Bio-based Fillers	CNC/CNF	Mechanical reinforcement Flame retardancy	TEMPO oxidation Acetylation
